# Important hemoprotozoan diseases of livestock: Challenges in current diagnostics and therapeutics: An update

**DOI:** 10.14202/vetworld.2016.487-495

**Published:** 2016-05-20

**Authors:** Biswa Ranjan Maharana, Anup Kumar Tewari, Buddhi Chandrasekaran Saravanan, Naduvanahalli Rajanna Sudhakar

**Affiliations:** 1 Department of Veterinary Parasitology, College of Veterinary Science and Animal Husbandry, Junagadh Agricultural University, Junagadh, Gujarat, India; 2 Division of Parasitology, Indian Veterinary Research Institute, Izatnagar, Uttar Pradesh, India

**Keywords:** *Anaplasma*, *Babesia*, chemotherapy, hemoprotozoa, molecular diagnosis, *Theileria*, *Trypanosoma*

## Abstract

Hemoprotozoan parasites pose a serious threat to the livestock population in terms of mortality, reduced milk yield and lowered draft power. Diagnosis of these diseases often poses a challenging task. Needless to say that impact of disease in health and productivity is huge though a fair economic assessment on the quantum of economic loss associated is yet to be worked out from India. The diagnosis of hemoprotozoan infections largely depends on various laboratory-based diagnostic methods as the clinical manifestations are often inconspicuous and non-specific. Traditional diagnostic methods rely on microscopical demonstration of infective stages in blood or tissue fluids. However, it is laborious, lesser sensitive, and cannot differentiate between morphologically similar organisms. Recent development in the technologies has opened new avenues for improvement in the accurate diagnosis of parasitic infections. Serological tests are simple, fast but lack specificity. With advent of molecular techniques, as DNA hybridization assays, polymerase chain reaction and its modifications ensure the detection of infection in the latent phase of the disease. Nucleic acid-based assays are highly sensitive, free from immunocompetence and can differentiate between morphologically similar parasites. With the advent of newer diagnostics complemented with traditional ones will be of huge help for targeted selective treatment with better chemotherapeutic agents.

## Introduction

Protozoan parasites are responsible for causing severe infections both in humans and animals worldwide. The infection is mainly transmitted by arthropod vectors, or through blood transfusion [1]. The important hemoprotozoan diseases of veterinary importance are trypanosomosis, theileriosis, babesiosis, and anaplasmosis, which are caused by several species of *Trypanosoma* [2], *Theileria*, *Babesia* [3-5], and *Anaplasma*, respectively, in several species of livestock. The impact of diseases caused by these organisms on health and productivity of farm animals and human beings is huge, though a fair economic assessment on the quantum of incidental economic loss is yet to be worked out from India [6]. The clinical manifestation of the disease varies from fever, anorexia, anemia, threatened abortion, and death in the acute form of infections [7]. Conventional diagnostic methods for most of the infections rely on microscopical demonstration of infective stages in blood or tissue fluids. Diagnosing hemoprotozoan infections by light microscopy examination of invasively acquired specimens at chronic stage of infection is a challenging task. Serological tests developed and designed for indirect antibody-based diagnosis of the infectious parasitic diseases during the year 1970s had wide acceptance globally though a specific serological reference test for many of the infections are still to come. The major difficulty associated with standardization of such test is the paucity of specific non-cross-reactive test reagents [8]. Most popular serologic assays used to detect antibodies to most of the hemoparasitic infections in test sera from animals and humans include immunofluorescence, enzyme-linked immunosorbent assay (ELISA) and its variants and Western blot. The sensitivity and specificity of these tests for detecting infection specific antibody are not known because few comparative evaluations have been published. Detecting some pathogens in infected cell cultures may be possible, but isolating the organisms in cell culture as a means of diagnosing infection is laborious and lengthy and is prone to failure with specimens from non-sterile sites. Therefore, the cell culture is not recommended as a routine laboratory technique for diagnosing most of the hemoparasites hence, though animal inoculation test is ethically prohibitive, still a dependable diagnostic strategy for blood-borne parasites such as trypanosomes. Keeping these in mind, nowadays, molecular diagnosis is the method of choice for identification of parasites. Commonly used molecular tools are polymerase chain reaction (PCR), real-time PCR, loop-mediated isothermal amplification (LAMP), etc. [9]. The nucleic acid-based techniques have the advantage of ensuring the detection of infection in the latent phase of the disease when the level of parasitemia is often below the detection limit of conventional methods as well as in the assessment of the success of the specific chemotherapeutic intervention, besides its epidemiological applications [10]. Although total eradication of the protozoan infections may be impossible, strategies for effective control of the infections in many tropical countries like India can be a feasible goal. To ensure this goal, application of techniques with proven impact needs to be applied especially for identifying the carrier animals. Ironically, diagnostic decisions still rely on microscopy ostensibly for economic reasons. Compared to the microscope and serology-based techniques, the molecular methods offer more sensitivity and specificity, which greatly increase the probability of specific detection in test samples. The molecular detection techniques are useful even in dealing with a large number of samples at a much higher sensitivity and have the required flexibility of automation and up gradation [10]. Therefore, the techniques can be applied for the prevalence studies of a disease of veterinary and zoonotic importance.

### Trypanosomosis

Trypanosomosis popularly known as surra is one of the most important hemoprotozoan diseases affecting human and animal health in the tropics [11,12] especially in the South East Asia [13]. The prevalence of surra peaks around the monsoon when the animals are under maximum work-stress owing to agricultural plantations, besides other contributing factors, *viz*., concurrent disease, poor nutrition, innate and acquired resistance, parasite pathogenicity, and strain of parasite. Effective surveillance for *Trypanosoma evansi* is constrained by a lack of sensitive diagnostic tests and information on *T. evansi* distribution in India. Although the level of awareness in veterinarians and diagnostic laboratories with regard to *T. evansi* is almost adequate, lack of diagnostic techniques of high sensitivity hinders quick identification. Microscopy is the only diagnostic tool besides animal inoculation with virtues of high specificity, ease of use, lack of cold chain [14]. However, its low sensitivity (approximately 100,000 parasites/ml for wet blood film examination, leads to an erroneous judgment of false negativity which often results in even death of the infected individual in the absence of treatment [15]. Only with concentration methods such as microhematocrit centrifugation [16], quantitative buffy coat technique [17], and mini-anion-exchange centrifugation technique [18,19] can detect parasitemia as low as 50 parasites/ml. This limits the utility of microscopy in resource-poor settings.

Immunodiagnostic techniques are useful for diagnosis of trypanosomosis, but most of them have been retrospective surveys so ineffective in controlling the ailment. However, the complement fixation test was used successfully in the control and eradication of dourine in North America [20] and the diagnosis of surra in buffalo in the Philippines [21]. The indirect fluorescent antibody test (IFAT) is both specific and sensitive for detection of trypanosome antibodies in animals and humans [22]. However, a major problem associated with IFAT is the cross reactivity between different species of *Tryponosoma* and requirement of the sophisticated microscope. Other immunodiagnostics followed very often are ELISA [23], card agglutination test (CATT), etc. [24]. CATT can detect early infections as it can detect immunoglobulin IgM. On the contrary, ELISA used to detect immunoglobulin IgG in established infections. A monoclonal antibody-based latex agglutination test has been claimed effective in the diagnosis of surra in domesticated animals under field conditions, which have been reported to be simple, rapid, and cost effective in field conditions [25-27].

A range of nucleic acid amplification-based molecular techniques has been developed to improve the detection of pathogenic trypanosomes. Among them, PCR has the maximum application [28,29]. Several PCR-based diagnostic assays have been developed which include the use of species-specific primers, single and nested PCRs [30], and real-time PCR [31]. Tandem repeat domain of GM6 (cytoskeletal protein) has diagnostic value and its usefulness as seroepidemiological studies of surra has been evaluated among water buffaloes [32]. PCR based on invariant surface glycoprotein 75 gene can be useful in the detection of carrier status of surra in animals [33]. With the use of generic primers in semi-nested PCR targeting variable region of 18s rDNA gene followed by the restriction fragment length polymorphism (RFLP) approach, it is possible to differentiate between important trypanosome species infecting bovines with mixed infections [34]. However, requirements for trained manpower and sophisticated equipment have restricted its use for wide application in the endemic areas. Isothermal reactions, such as LAMP [35] and nucleic acid sequence-based amplification (NASBA) [36,37], have recently been developed for the diagnosis of trypanosomosis; however, the techniques have increased application only for detection of human cases. These diagnostic tests have advantages that they do not need expensive equipment, post-amplification handling requirements and also are highly sensitive in terms of detection of pathogen [38]. However, the importance of PCR in the control of trypanosomosis in screening samples from serologically positive field samples in a specialized reference laboratory and the high accuracy would ease early detection and epidemiological mapping of trypanosomosis. More recently, proteome (i.e., the whole parasite content) and secretome (i.e. naturally excreted/secreted molecules) analysis providing valuable clues for identification of immunodominant antigens of *T. evansi* for detection and control of chronic trypanosomosis [39].

### Chemotherapy

Diminazene aceturate (DA) is the most extensively used curative trypanocide against surra in ruminants followed by isometamidium chloride (IMC) (both curative and preventive), cymelarsan (for curative treatment of camels), suramin, and quinapyramine (curative and/or preventive). Its use in horses and dogs is limited due to poor efficacy and tolerance in these species. IMC, a member of phenanthridine family, can be used for curative (0.5 mg/kg bw) and preventive (1 mg/kg bw) treatment of surra in ruminants and horses *via* intramuscular or subcutaneous injection [23]. Further, DA and IMC constitute a “sanative pair,” which means that once resistance develops to one of the drugs and another drug can be used to control the infection. Melarsominedihydrochloride (cymelarsan) is the latest trypanocide to be discovered. It is used to control surra at a dose rate of 0.25, 0.25-0.5, 0.5, and 0.75 mg/kg bw in camels, horses, cattle, and buffaloes, respectively. Quinapyramine methyl-sulfate, a member of aminoquinaldine derivatives, can be used to treat the infection by subcutaneous injection at a dose of 5 mg/kg bw. A more effective mixture of quinapyramine chloride and quinapyramine sulfate (triquin) can be used as a curative/preventive drug against *T. evansi* in horses and camels administered by subcutaneous injection at a dose of 8 mg/kg bw. The drug has chemoprophylactic effect which can last up to 4 months. Its use should be restricted to horses and camels only. Quinapyramine is not recommended in cattle because it may induce cross resistance to both IMC and DA [23].

The 50% inhibitory concentrations of the four existing agents were reported as 87.6, 12.5, 1.1, and 0.1 ng/ml for suramin, diminazene, cymelarsan, and quinapyramine, respectively. The diamidine diminazene reported to have the least efficacy among all of the standard drugs currently available in the market. In contrast, cymelarsan demonstrated the best efficacy, yet the costs of this drug, which is higher than those of other trypanocidal drugs, coupled with its limited availability throughout the world (it is available only in the Middle East and Africa), overshadow its beneficial properties. Variation of the glycoprotein between and within *Trypanosoma* spp. is a major stumbling block for vaccine development against this parasite. Hence, researchers are in the process of identifying non-variant regions such as PFR protein, which is universally present in the kinetoplastid flagellum as potential drug and vaccine target [40-44]. Against the backdrop of failed attempts to develop a protective vaccine using native or recombinant proteins of trypanosome origin, development of a live attenuated vaccine using ionizing radiation is believed to be impressive and practical. We are presently working in this area to explore the immunoprophylactic potential of irradiated *T. evansi* in a murine model. The radiation attenuated *T. evansi* was used in immunization trial involving rats and mice. The immunized rats and mice could withstand a lethal homologous challenge, and the conferred protection was attributed to both the humoral and cellular immune responses. Based on the encouraging response achieved through irradiation attenuated parasites in the preliminary studies involving rodent model, an experimentation involving the target bovine model may be emphasized further as it has shown promise toward the development of a live attenuated vaccine against surra (Unpublished Data).

### Babesiosis

Babesiosis is caused by hemotropic protozoa belonging to the genus *Babesia*, family Babesiidae and order Piroplasmida, within the phylum Apicomplexa. This protozoan parasitizes the erythrocytes of wild and domestic animals. The infection has long been recognized as an economically important disease of cattle, horses, and dogs and has gained increasing attention as an emerging zoonotic disease. Bovine babesiosis or red water fever is prevalent in cattle worldwide [45]. In India, *Babesia bigemina* is the important species infecting cattle. Clinical signs characteristic of infection in bovines are increased in body temperature, high rate of pulse, and respiration with a marked decrease in appetite and disinclination to movement. In acute infection, hemoglobinurea and hemolytic anemia are characteristic with fatal outcome in absence of chemotherapy [46].

Traditionally, the microscopic detection of *Babesia* parasites has always been considered as the gold standard for the diagnosis of acute babesiosis [47]. However, the low sensitivity of the technique is the major drawback which makes it difficult to detect low parasitemia in the chronic stage of infection as well as in the carrier animals [48,49]. Serological test-like IFAT, due to their better sensitivity, is considered as a suitable protocol for diagnosis of infection [50], but cross reactivity among species and also in genus level is really a major drawback for species-specific diagnosis. Nucleic acids-based detection methods developed in recent past with increased specificity and sensitivity. PCR-based assays have been widely used for the detection of *Babesia* parasites owing to their high specificity and sensitivity [51,52]. PCR enables detection of *Babesia* parasitemia as low as a few organisms per milliliter. Real-time PCR is useful, especially in early stage of infection when results of serological tests are negative, and the blood smear does not reveal the pathogen [53,54] or where the distinction between inter-erythrocytic forms of related organisms, viz., *Theileria* sp. and *Plasmodium* sp. required. Reverse line blot (RLB) is a more sensitive method than PCR since it is able to detect extremely low parasitemia levels and simultaneously identify *Theileria* and *Babesia* species using specific oligonucleotide probes [55]. The utility of LAMP technique for diagnosis of babesiosis infection in cattle has been successfully demonstrated [56]. NASBA or transcription-mediated amplification or self-sustaining sequence replication system, which is a highly sensitive, isothermal, transcription-based amplification system specifically designed for the detection of RNA targets. NASBA employs a battery of three enzymes, i.e., RNAase H, reverse transcriptase and T7 DNA dependent RNA polymerase, and two primers leading to main amplification product of single-stranded RNA. It is recently used for diagnosis of *Babesia* and *Theileria* using RNA as an initial template [57].

The latest development in molecular biology has created exciting possibilities for improved specific diagnosis of hemoprotozoan diseases. The 18S rDNA gene (18s rDNA, SSU rDNA) encoding rRNA of the small ribosomal subunit is one of the frequently used molecular markers in diagnostic and epidemiological studies. In *Babesia*, the gene encoding 18s rRNA harbors eight variable regions numbered V_1_ to V_5_ and V_7_ to V_9_ among which the V_4_ gene (300 bp) is the biggest and most changeable region. Nested PCR enables the amplification of this region characteristics for certain *Babesia* species or even strains within this species. With the aim of developing the molecular diagnostics of babesiosis, Birkenheuer *et al.*, 2003 carried out semi-nested PCR to detect and differentiate the DNA of *Babesia* *gibsoni*, *Babesia* *canis*, and *Babesia* *vogeli* in canine blood samples [58]. It is emphasized that in the diagnosis of babesiosis, the determination of the species, sub species, and even genotype that caused the babesiosis is very essential as virulence, prognosis, and response to treatment against *Babesia* species is different. Molecular markers such as HSP-70, β tubulin allow the precise identification of *Babesia* species with the help of PCR-RFLP technique. Recently, PCR assay targeting *B. bigemina* small subunit ribosomal RNA has been used for detection of low-level *B. bigemina* infection in yaks at the yak rearing tracts of Himalayas [59]. Microchip electrophoresis (ME) using programed field strength gradient allows the analysis of 591 and 1191 bp DNA fragments from the 18 s RNA of *B. gibsoni* and *Babesia* *caballi* [60]. However, none of these nucleic acid-based methods could be considered better than another. Their utility in diagnostic applications varies as per the need.

### Chemotherapy

For chemotherapy of babesiosis three babesiacides, *viz*., quinuroniumsulfate (Ludobal^®^, Bayer Ltd.), amicarbalideiso-thionate (Diampron^®^, May and Baker Ltd.), and DA (Berenil^®^, Hoechst Ltd.) were available in most European countries, whereas only the last one is available in India. Diaminazine works rapidly against *Babesia* *bovis* and *B. bigemina* at a dose rate of 3.5 mg/kg intramuscularly and well tolerated. It will protect cattle from the two diseases for 2 and 4 weeks, respectively [61]. In the 1970s, a fourth, imidocarbdipropionate was introduced (Imizol^®^; Schering-Plough) for chemotherapy of red water fever. Imidocarb dipropionate rapidly became the product of choice because of its prophylactic efficacy at twice the therapeutic doses. Imidocarb is used subcutaneously at 1.2 mg/kg for treatment while 3 mg/kg provides protection from *B. bovis* for 4 weeks and *B. bigemina* for at least 2 months. At high dose, imidocarb also eliminates *B. bovis* and *B. bigemina* from carrier animals [45]. Imidocarb appears to be the drug of choice for treatment of infected horses (at 2 mg/kg, 4 mg/kg for *B. caballi* and *Babesia* *equi*, respectively). Later quinuronium and amicarbalide were withdrawn because of safety issue and diminazene, which is still widely used in the tropics for the chemotherapy of babesiosis and trypanosomosis was withdrawn from Europe for marketing reasons [62]. In addition, supportive therapies, such as blood transfusions, anti-inflammatory drugs, tick removal, iron reparations, dextrose, vitamins (B complex), purgatives, and fluid replacements, may be necessary in severe cases of babesiosis [63].

### Theileriosis

Bovine tropical theileriosis is a tick-borne infection caused by *Theileria annulata*, an intracellular protozoan parasite. It is a lymphoproliferative disease with high mortality and morbidity in cattle. *Theileria sergenti/buffeli/orientalis* cause mild or asymptomatic disease in cattle and well known as bovine benign theileriosis. Certain *Ixodid* ticks, such as *Hyalommaanatolicumanatolicum, H. m. marginatum*, and *H. a. excavatum* known to transmit *T. annulata*, are found in the Mediterranean region, especially in semi-arid areas [64]. Ticks of the genera *Amblyomma, Rhipicephalus*, and *Haemaphysalis* were suggested as a possible vector in the transmission of benign *Theileria* species [65].

Subclinical infection in cattle with *T. annulata* in endemic regions produces chronic carrier state and serves as sources of infection for ticks. Therefore, latent infections are important in the epidemiology of theileriosis [66]. The diagnosis of *Theileria* infection is based on clinical findings, and microscopic examination of Giemsa-stained thin blood and lymph node smears in acute cases. However, expertise in microscopy detection of piroplasm is required in subclinical or chronic infections because parasitemia is often extremely low and may otherwise be missed. Serological tests such as IFAT though provides adequate sensitivity and easy to perform but due to reduced specificity are not followed now a days. ELISA using recombinant proteins of *T. annulata* surface protein (TaSP) and *T. annulata* merozoite surface antigen 1 (TamS1) are being used to detect antibodies in infected animals [67]. A lateral flow device has been developed with recombinant TaSP antigen of *T. annulata* which did not show any cross reaction with hemoparasites of cattle [68]. ELISA based on 53 kDa recombinant protein from truncated EMA-2 gene of *T. equi* is able to detect *T. equi* specific antibodies as early as 9 days post-infection [69]. In recent time, PCR has been the most preferred method for detection of *Theileria* species in epidemiological studies. Several studies documented that PCR is more sensitive and specific than other conventional diagnostic techniques in determining piroplasm-carrier animals [70]. Different variants of PCR such as nested PCR [71] and real-time PCR [72], can be followed for fast and accurate diagnosis of the infection. ME is an increasingly popular technique due to its economical, time-saving nature and used successfully in the diagnosis of bovine theileriosis after amplifying the 816 bp DNA using only 200 nl of blood [73]. ME using programed field strength gradient allows amplification of 816 bp DNA fragment from the 18s RNA of *T. buffeli* [60]. RLB micro array is a recently developed technique that uses oligonucleotide probe to detect and identify *Theleria* and/*Babesia* simultaneously that by specifically amplifying the rRNA gene of V4 hypervariable region of all *Babesia* and *Theileria* species [74]. The obtained PCR products are then hybridized to nitrocellulose membrane, onto which different species-specific oligonucleotide probes are covalently linked. This assay has been extensively used in epidemiological surveys in various countries [75].

### Chemotherapy

Parvaquone (2-cyclohexyl-3-hydroxy-1,4-naphthoquinone) and buparvaquone (2-(trans-4-t-butylcyclohexyl-methyl)-3-hydroxy-1,4-naphthoquinone) are the two important chemotherapeutic agents used for the treatment of theileriosis caused by *T. annulata* in cattle without significant side effects or relapse of disease. Buparvaquone at a dose of 2.5 mg/kg has a satisfactory therapeutic index and is more effective in the chemotherapy of *T. annulata* than parvaquone [1].

Reliable vaccines of known efficacy have been developed for *T. annulata*. Rakshavac-T - the schizont tissue culture vaccine is meant for prevention of theileriosis caused in crossbred and exotic cattle. Attenuated schizonts do not produce the clinical disease. Immunized cattle can withstand the attack of infected ticks for a period of 3-year. In areas where the vaccinated animals are constantly exposed to tick bites, the immunity is constantly boosted and hence the immunity is conferred for lifetime. Where the animals are maintained in tick free condition, revaccination in every 3 years is recommended. The prophylactic use of Rakshavac-T and chemotherapy with buparvaquone could be the most promising means of controlling theileriosis. Vaccination against *T. parva* is based on infection and treatment, in which cattle is given a subcutaneous dose of tick-derived sporozoites and a simultaneous treatment with a long-acting tetracycline formulation. Recovered animals demonstrate a robust immunity to homologous challenge, which usually lasts for the lifetime of an animal [76].

### Anaplasmosis

Anaplasmosis is also known as gall sickness is an infectious non-contagious rickettsial disease caused by *Anaplasma marginale*. This is an obligate intra-erythrocyte rickettsial organism. It spreads through tick bites or by the mechanical transfer of fresh blood from infected to susceptible cattle from biting flies or by blood-contaminated fomites. The infection is also occasionally passed from an infected cow to her unborn calf through the placenta. Bovine anaplasmosis occurs in tropical and subtropical regions mainly due to *A. marginale* and *Anaplasma centrale*. Although cattle of all ages are prone to infection, adult cattle are more susceptible to infection than calves. It is worthy to note that recovered animals from primary attack remain as lifelong carriers [77]. The wild ruminants particularly cervids have been implicated as important reservoirs of infection for *A. marginale*, *Anaplasma Phagocytophilum*, and *Anaplasma ovis*. It infects the red blood cells, and the disease is characterized by fever, severe anemia, jaundice, brownish urine, loss of appetite, dullness or depression, rapid deterioration of the physical condition, muscular tremors, constipation, and pale mucous membrane and labored breathing [78].

Similar to babesiosis, diagnosis of bovine anaplasmosis can be made by microscopic detection of *Anaplasma* parasites by a well-prepared thin smear free from foreign matter, as specks of debris can confuse diagnosis. This method is suitable for clinically infected animals during the acute phase of the disease, but it is not reliable for detecting infection in pre-symptomatic or carrier animals [79]. In these cases, the diagnosis is made by serological tests. Several immunological and molecular assays have been established to detect this rickettsia in carrier animals, where parasitemia is low. Among serological tests, competitive ELISA using a recombinant antigen recombinant major surface receptor protein 5 (MSP5), and MSP5-specific monoclonal antibody has proven very sensitive and specific for detection of anaplasma-infected animals [80]. However, due to the presence of similar B epitopes in anaplasma species, the available serological tests display cross reaction among different species [81]. Nowadays, nucleic acid-based diagnostic methods with high levels of sensitivity and specificity are used. It includes arrays of tools such as RLB [82], PCR [83], PCR-ELISA [84], semi-nested PCR [85], and real-time PCR [86].

### Chemotherapy

All *Anaplasma* species infections in cattle respond to tetracycline in the early stages of infection at a dose rate of 6-10 mg/kg body weight thrice in a day. Long-acting oxytetracycline sometimes preferred with a dose rate of 20 mg/kg body weight with a single dose. Chronic infection can be eliminated by administrating long-acting oxytetracycline at a dose rate of 20 mg/kg body weight with at least two injections with 7 days interval. Imidocarb is highly effective against *A. maginale* at a dose rate of 1.5 mg/kg body weight [77,87].

Presently, there are two commercially available vaccines against anaplasmosis in the United States. Anaplaz^®^ is the first anaplasmosis vaccine manufactured for cattle in the United States made by Fort Dodge. More recently, Mallinckrodt (later Schering-Plough) marketed a vaccine called Plazvax^®^. Both the vaccines protect the animal against anaplasmosis by similar mechanisms. Two injections given at 4-6 weeks apart with annual boosters are recommended. This is a killed vaccine, which will prevent the death loss but not the disease [88]. In rare cases, new calves born to vaccinated dams may develop an anemia and die. In such circumstances, the early detection of the parasite becomes paramount importance.

## Conclusion

Specific diagnosis of the etiological agent is most important for control of the hemoprotozoan infections [89]. Microscopy-based detection methods are still the cheapest and fastest methods for diagnosis though the techniques suffer from the limitations of sensitivity and specificity. Comparatively, newer immunological tools offer faster and higher throughput over the conventional methods [90]. Serological tests commonly used for diagnosis of hemoprotozoan diseases have become more popular because of their high sensitivity. However, the efficiency of serological tests depends on the application of specific parasite-derived diagnostic antigen molecules that are yet to be identified. Although serological tests can be used to detect circulating antibodies, cross reactivity with antibodies against other species of piroplasms have been documented by various workers [91]. Moreover, antibodies tend to disappear in long-term carriers, whereas piroplasms persist. So, animals with a negative serological test can infect ticks and be the source of infection for other animals. Another pit fall with the serological tests is that the antibodies can still be detected years after recovery even though the parasite is not present in the circulation. Most of the serological tests employ crude/native parasite antigen and/or polyclonal antiserum as test reagents which result in poor specificity and lack of uniformity. Therefore, the traditional methods have been complemented by the molecular ones.

Nucleic acid amplification-based detection methods are sensitive and reliable; they are fast and very specific. Variants of PCR such as randomly amplified polymorphic DNA (RAPD), amplified fragment length polymorphism (AFLP), amplification refractory mutation system-PCR [92], and RFLP are also useful for species-specific diagnosis, genotyping (DNA polymorphism) and hence, in epidemiological studies of parasites. RAPD able to differentiate species of *Trypanosoma* [93], *Theileria* [94], and *Babesia* [95]. Similarly, population genetics of *Trypanosoma* [96] is investigated following AFLP. PCR-RFLP was also used to genotype trypanosome [97], *Babesia* [98], *Theileria* [99], and *A. marginale* [100,101]. Although the techniques are performed using sophisticated equipment, new methodologies are being developed to perform the tests without the need of expensive apparatus. A large reservoir of asymptomatic infections has been detected using the molecular methods [102-104]. Most of the recently developed molecular techniques are amenable to high-throughput scaling up for larger sample sizes. These methods provide novel information on prevalence and epidemiology and are suited for active detection. The tools are also useful for sensitive molecular detection of carriers, especially in endemic areas. These advanced diagnostics complemented with well-controlled and efficient chemotherapeutic usage will provide the best means of controlling the hemoprotozoan infections and emergence of drug resistance.

## Authors’ Contributions

BRM collected and interpreted published information as part of his post-graduate thesis work. The manuscript was jointly prepared by BRM, AKT, and BCS. AKT and BCS as principal and co-guide of BRM during his post-graduate program incorporated valuable suggestions for improvement of the manuscript. BRM, AKT, BCS, and NRS drafted and revised the manuscript. All authors read and approved the final manuscript.
